# Small cell carcinoma of the urinary bladder: A case report and review of the literature

**DOI:** 10.3892/ol.2014.2646

**Published:** 2014-10-30

**Authors:** WEN-TING OU, QI-LIAN LIANG, XIN HUANG, ZHOU-YU LI, QIU-LONG LIU

**Affiliations:** 1 Department of Oncology, Affiliated Hospital of Guangdong Medical College, Zhanjiang, Guangdong 524001, P.R. China; 2 Department of Radiotherapy, Cancer Center of Guangzhou Medical University, Guangzhou, Guangdong 510095, P.R. China

**Keywords:** small cell carcinoma of the urinary bladder, dysuresia, painless gross hematuria, bladder

## Abstract

Small cell carcinoma of the urinary bladder (SCBC) is a type of rare malignant tumor of the urinary tract. As it does not have specific symptoms and its epidemiological features are similar to transitional cell carcinoma of the bladder, it is often misdiagnosed. SCBC is highly aggressive, metastasizes very early and has a poor prognosis, and consequently, it has become a focus for urological surgeons and oncologists. An 82-year-old male visited the Department of Urinary Surgery, in the Affiliated Hospital of Guangdong Medical College (Zhanjiang, China), due to gross hematuria that had persisted for one week. Abdominal computed tomography showed a neoplasm of ~6×6×7 cm on the anterior wall of the bladder. The initial diagnosis was of uroepithelial cell carcinoma of the bladder and surgery was performed to remove the tumor. However, the subsequent pathological examination suggested that the tumor was an SCBC. Small cell carcinoma is a highly malignant disease, with a high mortality rate, and it rarely occurs in the bladder. Upon review of a large number of studies, SCBC was not found to present with specific symptoms, making the early diagnosis of the disease difficult, however, commonly occurring symptoms included dysuria, painless gross hematuria and urinary tract obstruction.

## Introduction

Small cell carcinoma is also known as oat cell carcinoma. The disease has a high degree of malignancy, is not well differentiated from other diseases and has a poor prognosis. Small cell carcinoma mainly occurs in the lungs, but can also occur in other organs. Small cell carcinoma of the urinary bladder (SCBC) is extremely rare in the clinic, accounting for just 0.5–1% of all primary bladder tumors. The disease generally occurs in older males; the majority of patients develop painless gross hematuria (90%) and a few exhibit symptoms of bladder irritation. A minority of patients experience abdominal pain and urinary tract obstruction. Due to the low incidence of the SCBC, there are few clinical data-based prospective case-control studies. Therefore, it is difficult to establish a useful guideline for application in the clinic. It is common that SCBC is misdiagnosed as another tumor type, and is it therefore necessary to accumulate more experience to determine the best treatment plan. The present study reports the case of an 82-year-old male patient with SCBC whose diagnosis was confirmed by histopathological examination. The patient was treated by surgery following an initial diagnosis of uroepithelial cell carcinoma of the bladder, and written informed consent for the publication of this study was obtained from the patient’s family. The literature is also reviewed in the present study in order to aid in our understanding of the SCBC.

## Case report

An 82-year-old male was hospitalized in the Department of Urinary Surgery, in the Affiliated Hospital of Guangdong Medical College (Zhanjiang, China), on July 3, 2012, due to dysuresia and painlessness gross hematuria that had persisted for one week. The patient showed no signs of a fever, stomachache, diarrhea, coughing, expectoration or asthma. The medical history included a 10-year period of chronic bronchitis. Plain and enhanced pelvic computed tomography scans showed a neoplasm on the anterior wall of the bladder, indicating a carcinoma of the urinary bladder and possible prostate involvement. The neoplasm was removed on July 12, 2012, under general anesthesia. An extensive neoplasm was found on the posterior wall of the vesical vertex during the surgery. The neoplasm was cauliflower-like in appearance, ~6×6×7 cm in size and fragile. The results of the post-operative pathological examination of the neoplasm are as follows: i) The specimen conformed to the features of a neuroendocrine neoplasm and small cell carcinoma, and had invaded the full-thickness of the bladder, as well as the prostate and seminal vesicle. Local adenocarcinomic changes were present. ii) The incisal edges of the ureter and seminiferous ducts were free of cancer. The immunohistochemical assay showed positive staining for cytokeratin (CK)7 and CK20, partially positive staining for carcinoembryonic antigen and p63, weakly positive staining for synaptophysin, and no staining for thyroid transcription factor-1, chromogranin A (CgA), cluster of differentiation (CD)56, cancer antigen 125, P504S and prostate-specific antigen.

The patient did not undergo further treatment and was lost to follow-up, the ultimate cause of mortality is unknown.

## Discussion

Small cell carcinoma, a rare cancer with high malignancy and a high mortality rate, can occur in nearly all locations of the human body. However, it is most likely to develop in the lungs, and accounts for >20% of all lung cancers (1). SCBC is quite unusual, accounting for only 0.5–1% of all primary bladder carcinomas. Therefore, the pathogenesis of SCBC is currently unclear. There are a number of different theories explaining this pathogenesis, but the multipotential tumor stem cell theory and the neuroendocrine stem cell theory are the most dominant (2). Cheng *et al* (3) proved at the molecular genetics level that SCBC and transitional cell carcinoma originate from the same tissue cells. The study analyzed 20 cases (16 males and 4 females) of SCBC complicated by transitional cell carcinoma with loss of heterozygosity and X-chromosome inactivation (female patients), and found five polymorphic microsatellite markers and similar allelic loss modes of the two cancers in almost all patients. The general frequency of allelic loss was 90%. This was consistent with the multipotential tumor stem cell theory. Grignon *et al* (4) found that SCBC may originate from neuroendocrine stem cells of the bladder wall uroepithelium. Since SCBC often clinically co-exists with urothelial carcinoma and other histological cancers, the multipotential tumor stem cell theory is more popular than the neuroendocrine stem cell theory.

SCBC is quite similar to bladder urothelial carcinoma in terms of its symptoms, particularly hematuresis, which occurs at a rate of ~90%. Other common symptoms include dysuresia, urinary obstruction, chronic pelvic pain and urinary tract infection. Carcinoid syndrome, hypercalcemia and other syndrome inappropriate antidiuretic hormone secretions can also be observed occasionally (5). The present patient suffered from painless gross hematuria and dysuresia. SCBC does not have specific features with regard to clinical symptoms, signs or lab test results, so the diagnosis mainly depends on the pathological diagnosis and immunohistochemical assays. Typical microscopical features of SCC include small, round or oval-shaped tumor cells, a nest-like structure, little cytoplasm, hyperchromatic nuclei, a rough granular karyosome and frequently occurring massive mitotic figures, and extensive necrosis. The immunohistochemical assay is positive for CgA and CK, and negative for epithelial membrane antigen, leukocyte common antigen, synaptophysin and CD56 (6). Another characteristic is a lack of nest-like or glandular structures in adjacent tumor cells. Due to its high malignancy and low degree of differentiation, such cancer can easily invade and migrate to other tissues. By analyzing 642 SCBC patients, Koay *et al* (7) found muscular layer invasion in >90% of patients and lymphatic metastasis or distant metastasis in >80% of patients.

There is no defined treatment for SCBC. According to the experience of the Mayo Clinic (Rochester, MN, USA), a radical cystectomy should be applied to all SCBC patients unless there is metastasis (M1). Patients who have developed metastasis should undergo systemic chemotherapy. Chemotherapy is not necessary for T2 patients who have received radical cystectomy, but is essential for T3 and T4 patients (8). A number of studies (9–12) have shown that cystectomy itself cannot effectively treat SCBC and that a higher survival rate requires the use of chemotherapy and radiotherapy. Siefker-Radtke *et al* (13) from the MD Anderson Cancer Center (Houston, TX, USA) conducted a retrospective analysis of 46 SCBC cases and found that the combination of neoadjuvant chemotherapy and surgery can markedly increase the survival rate. The combined therapy provides a five-year survival rate of 78%, while the sole cystectomy treatment only imbues a rate of 36%. The study by Mangar *et al* (9) also supports the use of neoadjuvant chemotherapy based on previous clinical experience. Although pre-operative chemotherapy demonstrates useful effects, other studies indicate that post-operative chemotherapy is necessary despite the good effect provided by neoadjuvant chemotherapy (14). Currently, cisplatin, Adriamycin, cyclophosphamide and VP16 are common chemotherapeutic drugs. Combined chemotherapy is better than monotherapy. Common chemotherapy regimens include cis-platinum and etoposide (PE), and Adriamycin, vincristine and etoposide (11). Another study also describes the good outcome provided by methotrexate, vincaleukoblastine, Adriamycin and vincristine (15). Bastus *et al* (16) reported that subsequent to six cycles of alternate PE/ifosfamide, doxorubicin and vincristine, four out of five patients were free from tumor tissue under cystoscopy (1 succumbed during chemotherapy). With follow-up external-beam radiation therapy (bladder, 60 Gy; and pelvis, 45 Gy), one patient relapsed after 12 months, after surviving for 42 months post-surgery. The remaining three patients survived for 60, 48 and 27 months free of disease, respectively. This indicates that the combination of surgery, radiotherapy and chemotherapy should be applied to prolong survival time.

In conclusion, SCBC is a type of rare malignant tumor of the urinary tract that metastasizes early and has a poor prognosis, with a low degree of differentiation. The diagnosis can only be achieved by means of histological examination. Comprehensive therapy that combines surgery, radiotherapy and chemotherapy is the main method of treatment. Due to the low incidence of SCBC, there are no clinical data-based prospective case-control studies, therefore, more experience is necessary to analyze the best treatment plan.

## Figures and Tables

**Figure 1:**
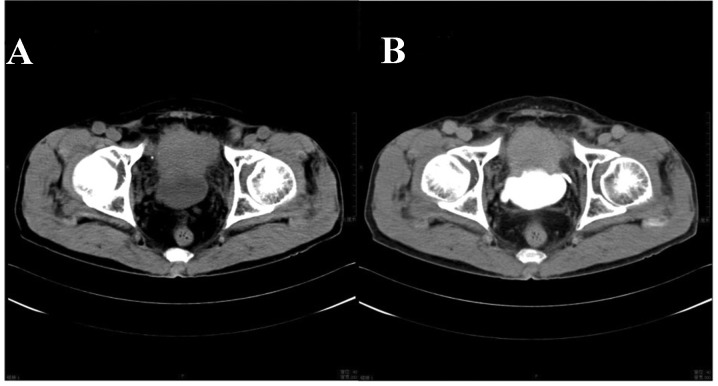
(A) Unenhanced computed tomography scan showing ~6×5-cm soft-tissue shadows measuring 44 HU in the front bladder wall. Irregular filling defects can be observed in the bladder. (B) Enhanced computed tomography scan showing poor definition between the prostate and nidus measuring 72 HU.

**Figure 2:**
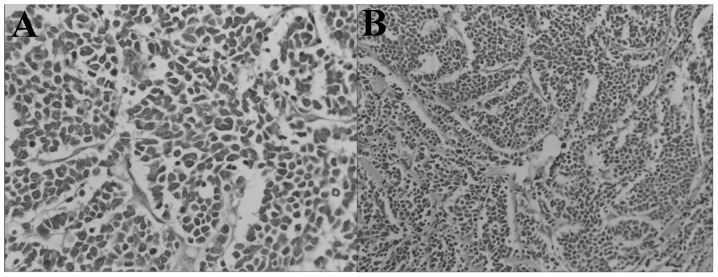
(A) The tumor showing that the nucleoli of the tumor cells had a high frequency of mitoses and an increased proportion of karyoplasm (original magnification, ×400). (B) The tumor showing a diffuse distribution or nested band arrangement (original magnification, ×100).
